# Protocol for a cluster randomised control trial evaluating the efficacy and safety of treatment for latent tuberculosis infection in recent migrants within primary care: the CATAPuLT trial

**DOI:** 10.1186/s12889-019-7983-7

**Published:** 2019-11-29

**Authors:** M. Burman, A. Copas, D. Zenner, V. Hickson, L. Gosce, D. Trathen, R. Ashcroft, A. R. Martineau, I. Abubakar, C. Griffiths, H. Kunst

**Affiliations:** 1 Institute of Population Health Sciences, Barts and The London School of Medicine and Dentistry, Queen Mary University of London, Yvonne Carter Building, 58 Turner Street, London, E1 2AB UK; 2 Institute for Global Health, University College London, London, UK; 3 Newham Clinical Commissioning Group, London, UK; 4 School of Law, Queen Mary University of London, London, UK; 5 Blizard Institute, Barts and The London School of Medicine and Dentistry, Queen Mary University of London, London, UK; 6 Barts Health NHS Trust, London, UK

**Keywords:** Tuberculosis, Public health, Respiratory medicine, Primary care

## Abstract

**Background:**

The identification and treatment of LTBI is a key component of the WHO’s strategy to eliminate TB. Recent migrants from high TB-incidence countries are recognised to be at risk TB reactivation, and many high-income countries have focused on LTBI screening and treatment programmes for this group. However, migrants are the group least likely to complete the LTBI cascade-of-care. This pragmatic cluster-randomised, parallel group, superiority trial investigates whether a model of care based entirely within a community setting (primary care) will improve treatment completion compared with treatment in specialist TB services (secondary care).

**Methods:**

The CATAPuLT trial (Completion and Acceptability of Treatment Across Primary Care and the community for Latent Tuberculosis) randomised 34 general practices in London, England, to evaluate the efficacy and safety of treatment for LBTI in recent migrants within primary care. GP practices were randomised to either provide management for LTBI entirely within primary care (GPs and community pharmacists) or to refer patients to secondary care. The target recruitment number for individuals is 576. The primary outcome is treatment completion (defined as taking at least 90% of antibiotic doses). The secondary outcomes assess adherence, acceptance of treatment, the incidence of adverse effects including drug-induced liver injury, the rates of active TB, patient satisfaction and cost-effectiveness of LTBI treatment. This protocol adheres to the SPIRIT Checklist.

**Discussion:**

The CATAPuLT trial seeks to provide implementation research evidence for a patient-centred intervention to improve treatment completion for LTBI amongst recent migrants to the UK.

**Trial registration:**

NCT03069807, March 2017, registered retrospectively.

## Background

TB remains the leading cause of death among infectious diseases globally [1]. The WHO strategy to eliminate TB prioritises screening and treatment of those at high risk of latent tuberculosis infection (LTBI) [2]. It is estimated that approximately a quarter of the world’s population (1.7 billion people) have LTBI [3]. Epidemiological modelling indicates that without successful treatment of LTBI in addition to active case finding it will be impossible to eliminate TB by 2050 [4] .

Recent migrants from high TB-incidence countries are recognized to be a group with a high LTBI prevalence and risk of TB reactivation [5]. In many high-income countries, where a high proportion of TB cases occur amongst the foreign-born, TB strategies have focused on LTBI screening and treatment programmes for this group, including England, which in 2015 made the systematic screening and treatment of LTBI in migrants a core component of its national TB control strategy [6]. However, traditional models of care are associated with low rates of completion in the LTBI cascade-of-care, with recent migrants recording the lowest rates of any at-risk group [7]. Recognising the weaknesses of traditional models of care, the End TB Strategy makes “integrated, patient-centred care and prevention” a pillar of its action-frame work for elimination and calls for urgent “research to optimise implementation” of novel strategies [2].

The London borough of Newham has been a pilot site for the national LTBI screening programme for recent migrants since 2014. Newham has the highest incidence of active TB in the United Kingdom (UK), with 86% of cases in 2014 occurring in those born outside the UK [8]. The programme in Newham has adopted a novel model of care for treating LTBI that occurs entirely within the community: GPs offer screening with an IGRA. Patients with positive results are assessed and if diagnosed with LTBI offered treatment that is monitored by a trained community pharmacist in collaboration with the patient’s GP. This is the first time in the UK that LTBI has been managed systematically outside of a specialist TB service. The proposed national model for LTBI screening in England is to screen for LTBI in primary care but for those identified with LTBI to be referred for assessment and treatment to secondary care (specialist TB services) [9]. Specialist TB services for Newham are provided by the TB clinic at Newham University Hospital, Barts Health NHS Trust.

We will conduct a pragmatic cluster-randomised, parallel group, superiority trial to evaluate the efficacy and safety of treatment for LTBI in recent migrants within primary care as compared to treatment in secondary care. The primary objective will be treatment completion (defined as taking at least 90% of antibiotic doses on pill count). The secondary objectives of the trial will assess adherence, acceptance of treatment, the incidence of adverse effects including drug-induced liver injury (DILI), the rates of active TB, patient satisfaction and the cost-effectiveness of treatment.

## Methods and design

### Study design

This is a pragmatic cluster-randomised, parallel group, superiority trial of at least 20 randomisation units (20 of 60 GP Practices) in the London borough of Newham, UK.

The cluster-randomised design was chosen because the delivery of LTBI management in primary care provided by multiple health care professionals across various locations occurs at the level of the cluster. The use of individual randomisation was not feasible because it would disrupt the delivery of a complex intervention at participating GP practices. This protocol follows the SPIRIT guidelines [10].

### Setting/intervention

The trial compares two models of care for LTBI treatment: management in the community by a GP and community pharmacist (intervention) or in secondary care by a TB doctor and TB nurse (control). The setting will be the London borough of Newham.

Since 2014, all GP practices (*n* = 60) in Newham have been contracted to screen and treat recent migrants for LTBI as a pilot site for the national LTBI screening and treatment programme. Eligible patients are those aged 16–35 years, from a country with a TB incidence > 150 per 1,000,000 per year or sub-Saharan Africa, who have arrived in the UK within the last 5 years. The CATAPuLT study protocol mirrors the existing service specification for the Newham LTBI programme. Nationally the model for LTBI care in recent migrants is to screen in primary care and to refer those with positive results to secondary care for assessment and treatment.

In the CATAPuLT trial, all GP practices will continue to screen eligible patients for LTBI. Practices will be randomised to either provide assessment and treatment for LTBI in primary care (the intervention arm) or to refer patients to secondary care (the control arm, which represents the current standard of care). The test for LTBI is an interferon gamma release assay (IGRA).

To confirm a diagnosis of LTBI, any patient with a positive IGRA must be reviewed by a doctor to exclude active TB. The medical assessment includes a history, physical examination, a chest radiograph and blood tests (full blood count, liver function tests, urea and electrolytes, c-reactive protein and blood-borne virus serology (Human Immunodeficiency Virus (HIV), Hepatitis B Virus (HBV) and Hepatitis C Virus (HCV)). Those diagnosed with LTBI are offered treatment with 3 months of Rifinah®, combination rifampicin and isoniazid therapy (Sanofi, UK), and pyridoxine.

In the intervention arm, the GP excludes active TB, and offers treatment for LTBI. The patient is asked to select one accredited community pharmacy for treatment. The borough has 16 community pharmacies that have been trained and accredited to provide LTBI treatment. Treatment is initiated and monitored by a community pharmacist. At their initial visit, the community pharmacist will counsel the patient about LTBI treatment. The patient is then issued with a 1 month supply of medication and asked to attend for liver function tests (LFTs) after 2–4 weeks. The patient attends the pharmacy at the end of each of the 3 months of treatment to assess adherence and adverse effects. In the control arm, the TB clinician and TB nurse mirror the role of the GP and community pharmacist respectively. Prior to starting treatment, a questionnaire will be used to assess knowledge about LTBI.

Follow up is identical in trial both arms: at the end of each month of treatment adherence is assessed using a pill count (or self-report if the patient fails to attend with their tablets), and the Medication Adherence Report Scale (MARS-5), a set of 5 statements about common patterns of adherence rated on a 5-point Likert scale (1 = always, 2 = often, 3 = sometimes, 4 = rarely, and 5 = never) [11]. At the first and second visits, urine is tested for metabolites of isoniazid (Isoscreen®, GFC Diagnostics, Oxfordshire, UK). At the second visit, the patient completes a satisfaction questionnaire. Prior to starting treatment, a questionnaire will be used to assess knowledge about LTBI. See Table 1.

**Table 1:** Timetable of assessments in the CATAPuLT Trial. For Urine testing a minimum of two urine tests should be performed during the 3 months of treatment. These should be done at the review visit for months 1 and 2 of treatment, although the second may be done at the final visit instead if the patient was unable to provide a sample at either of the earlier visits, or additionally at the final visit if there are concerns about adherence, *LFTs Liver function test, **INH Isoniazid

Assessment Tool	Day 0	Day 15	Day 30	Day 60	Day 90
Questionnaire (Understanding and knowledge of LTBI)	X				
Blood tests for LFTs*		X			
Pill count / MARS5 tool			X	X	X
Questionnaire (adherence/ adverse effects)			X	X	X
Urine test (Colour and INH **metabolites)			X	X	
Patient satisfaction questionnaire				X	

### Inclusion criteria

Patients identified with LTBI aged 16–35 and who have entered the UK within the last 5 years, from a country with a WHO-estimated annual TB incidence of ≥150 per 100,000 per year or sub-Saharan Africa. LTBI is defined as a positive IGRA test without symptoms, physical signs or radiological evidence of active TB.

Between October 2017 and April 2018, Newham CCG piloted extended eligibility criteria to include those who had been resident in the UK for up to 10 years. The trial protocol was amended to mirror this change.

### Exclusion criteria

- Pregnant or breastfeeding women.
- Patients requiring medications that cannot be safely taken with Rifinah
- HIV infection
- Individuals with known liver disease, or abnormal liver function tests (LFTs).
- Diagnosis of cirrhosis
- Chronic or active HBV or HCV infection.
- Previous treatment for TB or LTBI
- Individuals who are unable to consent or who would usually be offered LTBI treatment under DOT
- Evidence of active TB (based on history, examination, blood tests and/or chest radiograph).

These exclusion criteria match the service specification for the existing local LTBI programme. IGRA positive patients with complex co-morbidities or health needs are referred to specialist TB services for assessment and treatment.

### Outcomes

#### Primary outcome –treatment completion

Completion of at least 90% of prescribed therapy as assessed by pill count. Where a patient fails to bring their medication, information will be obtained by patient report. Treatment completion will be assessed at the final patient review, approximately 3 months after treatment is commenced. To avoid bias, an independent researcher, blinded to allocation, will verify treatment completion where there is uncertainty. This outcome is defined for IGRA positive patients who accept treatment.

#### Secondary outcomes

- Proportion of participants completing < 80, 80–89.9, ≥90% of antibiotic dosages based on pill count.
- Treatment adherence assessed using the five-point adherence questionnaire (MARS5 tool), prescription collection and a point-of-care urine testing for metabolites of Isoniazid (Isoscreen) at monthly intervals.
- Treatment acceptance assessed by calculating the proportion of eligible patients that accept therapy; this is defined as those initiating treatment and attending TB clinics and community pharmacies on at least one occasion.
- The number of patients on treatment having adverse events including DILI leading to discontinuation of treatment or hospitalisation. Safety of treatment will be assessed every month by the pharmacist in primary care and by the TB nurse in secondary care.
- The incidence of active TB occurring within 2 years after enrolment. TB incidence in the intervention and control group will be compared and there will be a sub-group analysis to evaluate the effect of the intervention in those who did or did not complete treatment. This will be performed through matching the study population with the national Enhanced TB Surveillance System, where information on all reported TB cases nationally are recorded.
- Assessment of patient satisfaction.
- Evaluation of cost-effectiveness of LTBI treatment in primary care compared to secondary care.

#### Additional outcomes

Identification of factors associated with treatment non-acceptance and completion using a standardised questionnaire to explore patients’ understanding and knowledge of LTBI.

### Sample size

We predict treatment completion in primary care to improve by 15% compared with secondary care (from 70 to 85%). To detect this difference with 80% power, and 5% significance level, an individually randomised trial would require 268 participants. We planned to conduct our trial in a minimum of 20 GP practices (10 intervention and 10 control), noting that as the number of GP practices increases, the required total sample size of individual participants falls. With 20 practices, we would have needed to adjust for the effect of clustering by increasing the sample size to 780 participants (or 39 patients per practice) assuming an ICC of 0.05. To allow for loss to follow up and treatment non-acceptance we would have inflated the required sample size by 30% to 1014 participants (51 per GP practice). The final number of practices randomised was 34, though as 14 were randomized after the initial phase we expect variability in cluster size reflected by a coefficient of variation of 0.5. Under the same assumptions as before the number of individuals required providing the primary outcome is 442 (221 per arm), inflated due to loss to follow up and treatment non-acceptance to 576 (17 per practice).

### Consent

Valid implied consent is assumed for participation, an approach used in two other recent trials in TB and HIV screening in London [12, 13] . All eligible patients are given a patient information sheet (PIS) in English and six other languages.

Eligible patients are asked for verbal consent to access their data which will be recorded in their electronic patient records. The PIS explains how patient data is collected and how patients may opt out of the study.

Our approach to consent was formally approved in our ethics application (see “Ethics approval”).

### Recruitment

It is expected that most eligible patients will be offered testing for LTBI at a “new patient check”, usually performed by a health care assistant or qualified nurse shortly after a patient registers with a GP practice. Patients will be given a PIS about the CATAPuLT trial when offered testing for LTBI.

The trial will also recruit eligible patients who are already registered with a GP. They will be given a PIS when offered an IGRA blood test. Eligible IGRA-positive patients tested prior to the trial opening who have not yet had a follow up appointment will be given a PIS and managed as per the arm of their GP practice. Eligible IGRA-positive patients tested prior to the trial opening who have had a follow up appointment but no prescription for treatment issued may be given a PIS and managed as per the arm of their GP practice. This group will be treated as a separate cohort and analysed separately from the main trial.

Cluster-level data about the number of positive tests recorded at each site will be monitored to ensure that all eligible patients are asked for consent to share their data with the trial team.

### Randomisation

Twenty GP practices were initially recruited and randomised, but after participant recruitment rates were lower than expected further practices were randomised across three further phases as practices became ready to join the trial. In the initial phase, 20 GP practices were stratified for randomisation by the cross-classification of two binary factors based on practice size and number of IGRA positives identified previously.

There were three further phases of randomisation for practices 21–22, 23–29 and 30–34 respectively. Of these, the randomisation of practices 23–29 was stratified by the number of IGRA positives and in the other phases randomisation was unrestricted. Practices 23–29 were randomised with the allocation of 3 to one study arm and 4 to the other (determined at random). In the final phase the allocation was 2 to one study arm and 3 to the other, allocating 3 to the arm that was allocated only 3 in the third phase, to ensure an overall 1:1 allocation of 17 practices to each arm. At all phases and within all strata the randomisation was implemented through random permutation using STATA v15 (STATA Corporation, College Station, Texas, USA). Randomisation was conducted by the trial statistician (AC). Information about study sites can be obtained from the corresponding author or chief investigator.

### Blinding

Data collection and analysis will be unblinded due to the nature of the intervention, cluster level randomisation and different data collection systems for the two study arms.

### Data collection methods and management

#### Study tools

Patient satisfaction and adverse effects will be assessed using non-validated questionnaires. Patient knowledge about LTBI will be assessed using a non-validated questionnaire adapted from two previous studies [14, 15]. The MARS-5 adherence questionnaire has not been validated for use in LTBI [11]. The Isoscreen® point of care test for metabolites of isoniazid (GFC Diagnostics, Oxfordshire, UK) has been validated in small prospective studies with a sensitivity of greater than 90% and a specificity of greater than 95% [16–18].

#### Data collection and management

Patient data will be entered electronically by the patients’ usual care team. All data collection will use purpose-built electronic management templates, adapted those developed for the national LTBI screening programme by the Clinical Effectiveness Group, Queen Mary University of London for Public Health England (PHE) [19]. Data will be managed and stored in accordance with local and national information governance specifications.

### Analysis

All analyses will be conducted on an intention-to-treat basis. The analysis will include all eligible participants regardless of how well the GP practice followed the study protocol or how well the participant complied with their treatment plan. The analysis will exclude any patients however who have withdrawn from the trial and specified they do not wish their data to be analysed.

Some outcomes are only applicable for certain subgroups. The primary outcome is defined only for patients who accept treatment. Patient satisfaction is only defined for patients who are still under care at 2 months, when the satisfaction questionnaire is administered. The MARS-5 and isoniazid metabolite urine testing adherence measures are only applicable at times when the patient is still taking treatment, i.e. not stopped due to adverse effects or by patient choice/withdrawal from the trial.

The primary outcome will be analysed using a logistic regression model. Results will be reported as odds ratios (OR) for the intervention compared to control with their corresponding 95% confidence intervals (CI) and two-sided *p*-values. The ICC for the primary outcome will be reported. Similarly, across the secondary outcomes treatment effect estimates will be presented with 95% confidence intervals based on regression models, using binary logistic regression, ordinal logistic regression or linear regression as appropriate. Regression models will include random intercept effects for GP practice.

Adjusted effect measures are considered the primary effect measures; unadjusted measures will also be reported. We will adjust the analyses for practice size and number of previous IGRA positives, our stratification factors, and selected pre-determined participant factors considered predictive of treatment completion.

### Missing data

Some patients will inevitably be lost to follow-up, arising primarily because they have withdrawn from treatment. For the primary outcome, a patient who withdraws from treatment without collecting all prescriptions will be classified as having failed treatment. We will impute missing data for those patients that collect all prescriptions but miss the final visit using multiple imputation by chained equations (MICE). A sensitivity analysis will also be performed in this group in which those failing to attend a final visit are assumed to have failed treatment.

### Health economic analysis

We will evaluate the cost-effectiveness of LTBI management in recent migrants within primary care settings as compared to secondary care settings using the trial cohort. A decision-tree model will be developed to compare the two strategies in terms of cost-effectiveness (cost/LTBI case treated). The methods of economic evaluation will follow the standard NICE reference case [20]. The analysis will be conducted from an NHS perspective with a two-year time horizon and standard discounting of costs and outcomes applied (3.5% per annum). The decision analysis model will describe the possible pathways of patients at the different time point of adherence control (t = 15, 30, 60, and 90 days) under each strategy: lost to follow-up, or ongoing treatment. Estimates of the costs of LTBI screening, LTBI treatment, management of adverse effects, and diagnosis of active TB will be obtained from the NHS Reference Costs [21]. Sensitivity analyses will be conducted to evaluate the impact of parameters on the results.

### Harms

The intervention in this trial is the setting of care. There are no specific anticipated harms associated with allocation within the trial. However, patients in both arms of the trial will be treated for LTBI with a 3-month course of daily Rifinah®. This preparation is licenced for use in the UK and is currently recommended for LTBI [20, 22].

Trial participants will be monitored for adverse events (AEs) and serious adverse events (SAEs) at each monthly review and are asked to undertake a blood test to assess liver function after 2 to 4 weeks of treatment.

### Ethics approval

Ethical approval for the trial was obtained from the Camden & Kings Cross Research Ethics Committee (REC), London, United Kingdom on the 11th March 2016. Protocol version and date**:** version 8, 13 August 2019.

### Patient and public involvement

Increasing patient choice and improving patient experience underlie the central hypothesis of the trial. The intervention enables patients to select an accredited pharmacy most convenient for them geographically and to attend outside office hours and at weekends in contrast to the specialist TB service. Prior to the design of the trial interviews were conducted with patients who had been treated for LTBI in the pilot service in Newham. The trial is supported by the chair of the “Patient Panel” at Barts Health NHS Trust and the local branch of Healthwatch, the independent consumer champion for health and social care in England. All questionnaires in the trial were piloted with eligible patients.

### Trial organisation

The trial will not have a data monitoring committee based on an assessment of risk (low). The trial will be overseen by a Trial Management Group (TMG) that will meet regularly until the end of the trial. The TMG will consist of the chief investigator, the trial team and co-investigators and collaborators.

### Study schedule and trial status

The first participant was enrolled in January 2017. The trial will remain open for data collection until December 2019.

### Dissemination

Trial results will be reported in scientific manuscripts for publication and disseminated through charities, stakeholder organisations and patient groups.

## Discussion

The WHO’s End TB Strategy and the Lancet Commission on TB both emphasise that there is an urgent need to make TB care patient-centred and that there is a lack of implementation research evidence to guide policy-makers in how to achieve this goal [2, 23]. It is also recognized that TB research must be “migrant-inclusive” [24]. This trial seeks to provide such evidence by evaluating the efficacy, safety and cost-effectiveness of a novel model of care for recent migrants to the UK with LTBI. We will assess whether this model of care results in higher rates of LTBI treatment completion. To our knowledge this is the only trial currently investigating novel models of care in the delivery of LTBI treatment. The results of the trial will inform national policy on the delivery of LTBI care to recent migrants in the UK but may be generalizable to other high-income settings and other groups with LTBI, such as recent contacts of active disease.

## Data Availability

Not applicable.

## Abbreviations

GP: General Practitioner
IGRA: Interferon gamma release assay
LTBI: Latent tuberculosis infection
TB: Tuberculosis
WHO: World Health Organization
